# Selection of Genetic Instruments in Mendelian randomisation studies of sleep traits

**DOI:** 10.1016/j.sleep.2023.10.036

**Published:** 2023-11-02

**Authors:** Valentina Paz, Hassan S. Dashti, Stephen Burgess, Victoria Garfield

**Affiliations:** a Instituto de Psicología Clínica, Facultad de Psicología, Universidad de la República, Tristan Narvaja 1674, Montevideo, 11200, Uruguay; b MRC Unit for Lifelong Health & Ageing, Institute of Cardiovascular Science, University College London, 1-19 Torrington Place, London, WC1E 7HB, UK; c Center for Genomic Medicine, Massachusetts General Hospital and Harvard Medical School, 185 Cambridge Street, Boston, MA 02114, USA; d Broad Institute, 415 Main Street, Cambridge, MA 02142, USA; e Department of Anesthesia, Critical Care and Pain Medicine, Massachusetts General Hospital and Harvard Medical School, 55 Fruit Street, Edwards 4-410C, Boston, MA 02114, USA; f MRC Biostatistics Unit, University of Cambridge, Forvie Site, Robinson Way, Cambridge CB2 0SR, UK; g Department of Public Health and Primary Care, University of Cambridge, Forvie Site, Robinson Way, Cambridge CB2 0SR, UK

**Keywords:** Genetic epidemiology, Sleep genetic variants, Review

## Abstract

This review explores the criteria used for the selection of genetic instruments of sleep traits in the context of Mendelian randomisation studies. This work was motivated by the fact that instrument selection is the most important decision when designing a Mendelian randomisation study. As far as we are aware, no review has sought to address this to date, even though the number of these studies is growing rapidly. The review is divided into the following sections which are essential for genetic instrument selection: 1) Single-gene region vs polygenic analysis; 2) Polygenic analysis: biologically- vs statistically-driven approaches; 3) P-value; 4) Linkage disequilibrium clumping; 5) Sample overlap; 6) Type of exposure; 7) Total (R^2^) and average strength (F-statistic) metrics; 8) Number of single-nucleotide polymorphisms; 9) Minor allele frequency and palindromic variants; 10) Confounding. Our main aim is to discuss how instrumental choice impacts analysis and compare the strategies that Mendelian randomisation studies of sleep traits have used. We hope that our review will enable more researchers to take a more considered approach when selecting genetic instruments for sleep exposures.

## Abbreviations

GWAS: Genome-wide association studies
IV: Instrumental variable
LD: Linkage disequilibrium
MAF: Minor allele frequency
MR: Mendelian randomisation
SNP: Single-nucleotide polymorphism
UKB: UK Biobank

## Introduction

Sleep is a complex phenotype regulated by homeostatic and circadian processes [1] and characterised by multiple dimensions such as duration, quality and timing or chronotype [2]. Studies have shown that these specific sleep traits are moderately heritable, with twin studies estimating that between 44-50% of their variability is genetically determined [3,4], while SNP-based heritability studies have shown that heritability of self-reported traits ranges from 5 to 15% [5–8]. Moreover, these dimensions have been consistently associated with several adverse health outcomes. For example, inadequate sleep duration, poor quality, and inappropriate timing are associated with adverse health consequences [9]. However, as most research has used observational epidemiology to study these associations, whether these links are causal remained elusive until very recently.

Mendelian randomisation (MR) is a method that uses genetic variants to assess causal relationships [10]. The MR method addresses two questions: whether an observational association between an exposure and an outcome is causal alongside the magnitude of this effect [11,12]. MR is increasingly used to overcome some limitations of traditional observational epidemiology, such as unmeasured confounding and reverse causality [10], and the analysis is facilitated by MR packages, such as the widely used “MendelianRandomization” package for the R open-source software environment [13] or the “mrrobust” Stata package [14]. Recently, numerous MR studies examined the causal relationship between genetic instruments of sleep traits and different health outcomes [11–56].

In the context of MR, a genetic variant can be considered an instrumental variable (IV) for a given exposure if it satisfies the following assumptions: i) it is associated robustly with the exposure of interest, ii) it does not influence the outcome through a pathway other than the exposure (horizontal pleiotropy) and iii) it is not associated with the outcome due to confounding [12]. Genetic variants used as IVs in MR are usually single-nucleotide polymorphisms (SNPs), a common variation at a single position of DNA sequence [10].

MR studies have steadily grown as genetic variants reliably associated with different exposures have increased over the last decade, thanks to genome-wide association studies (GWAS) [11]. GWA studies now test millions of genetic variants for their association with a given trait. Thus, finding genetic polymorphisms to use in an MR study is becoming more feasible. However, selecting optimal genetic instruments can be challenging [61].

**Figure 1:**
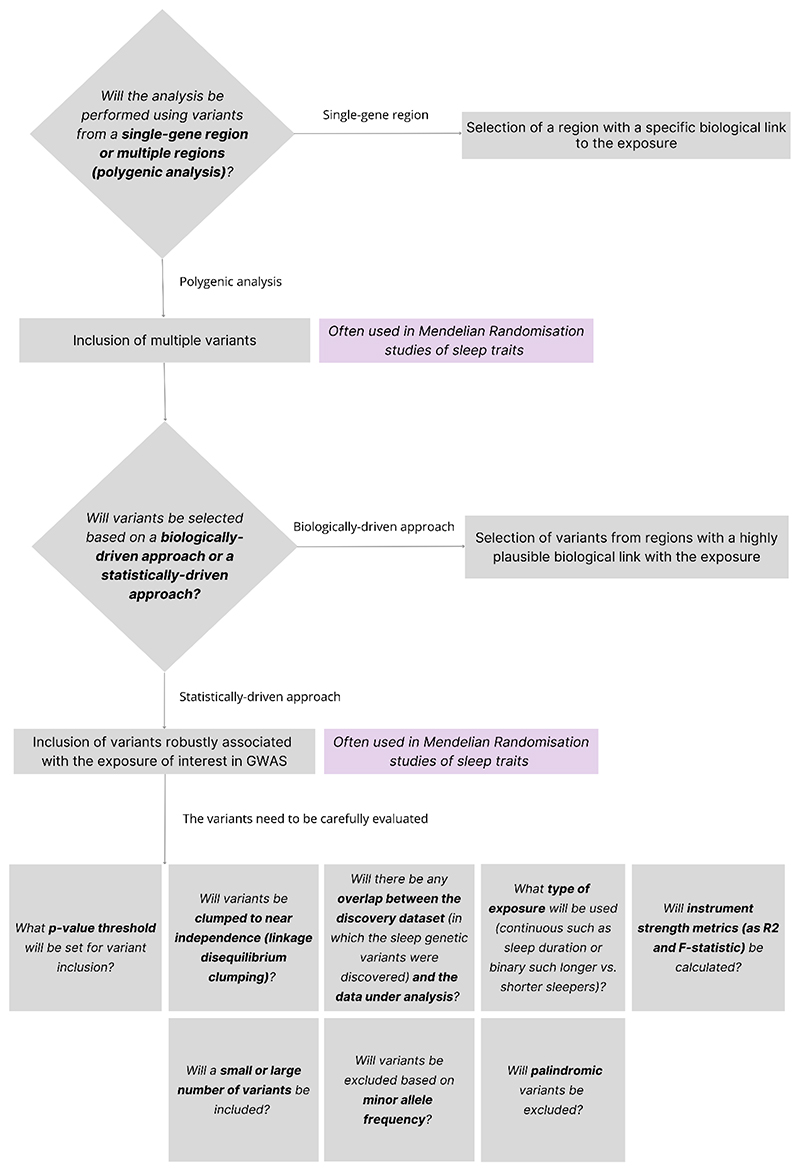
Flowchart with the main points to consider when selecting sleep genetic instruments in MR.

Although several guides exist for conducting MR studies [12,61,62], these are not widely adopted in the field of epidemiology of sleep; thus authors using genetic instruments for sleep traits have taken different approaches to the selection process. This review explores the criteria used for instrument selection in MR studies of sleep traits, discussing how this choice impacts analysis and some steps in the selection process that are often overlooked. We aim to demonstrate the importance of a careful selection of instruments to conduct an MR study. Nonetheless, it is worth mentioning that the selection process will always depend on the aim of the research and the specific exposure under study, and while we focus on MR studies of sleep traits, many of the issues discussed here apply to other behavioral phenotypes as well. In addition, even though MR has been particularly useful for understanding the causal role of sleep phenotypes on several health outcomes, other causal methods must also be used for replication and triangulation purposes. A summary of the main points to consider when selecting sleep genetic instruments in MR is presented in Figure 1.

## Criteria used to select genetic instruments in MR for sleep traits

### 1 Single-gene region vs. polygenic analysis

The first step in instrument selection is to decide whether the analysis will be performed using variants from a single gene region or multiple regions (a polygenic analysis). When a particular region has been reported to have a specific biological link to the exposure, the selection usually focuses on these variants [12]. This approach has the advantage of specificity, leading to a more plausible MR [63]. However, for complex risk factors such as sleep, no single gene region encodes this risk factor [64]. In fact, numerous genomic variants have been discovered by sleep GWAS in adults, indicating that sleep is a highly polygenic trait. For example, for insomnia, 554 risk loci have been reported in a recent study [65]. Thus, a polygenic analysis is often used in MR studies of sleep traits.

A polygenic analysis supposes the inclusion of multiple variants [12]. If the variants are all valid instruments, power is maximised because each SNP contributes incrementally to affect levels of the biomarker [61,63]. In the case of sleep, as common individual genetic variants confer small effects, the polygenic approach will typically have greater power to detect a causal effect than the single gene region approach [12].

### 2 Polygenic analysis: biologically-driven vs statistically-driven approach

For a polygenic analysis, one of two approaches may be chosen for selecting genetic variants: a biologically-driven or a statistically-driven approach [12]. The former implies selecting variants from regions with a highly plausible biological link with the exposure of interest [12,61]. The advantage of this approach is that these instruments may be less susceptible to horizontal pleiotropy [61]. However, biological understanding is rarely infallible [12], and the biological basis of sleep in humans is not fully understood [66]. Thus, instrument selection is often performed using a statistically-driven approach [67] or a combination of both approaches [12].

The statistically-driven approach exploits the increasing availability of SNPs associated with specific exposures in GWAS [61]. For this reason, authors tend to search for the latest and largest GWA study available and select SNPs robustly associated with the exposure of interest (MR assumption 1). However, it is important not to assume that the latest and largest study will always yield the best instruments. For example, most published GWAS of sleep traits have been performed in European samples and are also not sex-specific. Nonetheless, some GWAS have been performed in other ethnic groups, including Hispanic/Latino Americans [68] and multi-ancestry samples [69–74]. Furthermore, some have employed sex-stratified analyses for obstructive sleep apnea and insomnia, which display marked sexual dimorphism in disease prevalence [69,75]. However, further work is needed to better understand sex-related sleep differences, which have been associated with the influence of sex hormones on sleep regulation but have been understudied [76].

When using a statistically-driven approach, it is crucial to evaluate the reported SNPs carefully. Briefly, as described more thoroughly in the review, some important criteria for instrument selection include: 1) evaluating the number of variants to incorporate and their p-value, minor allele frequency (MAF) and whether they are palindromic; 2) selecting independent variants; 3) avoiding sample overlap between the discovery GWAS and the data under study (where possible); 4) prioritisation of GWAS with well-measured/defined phenotypes and determine whether to use a continuous or a binary exposure; 5) choosing variants based on their total and average strength and; 6) taking into account confounders of the genetic instrument-outcome relationship. If the instruments are not suitable, they could be selected from a different GWA study, which could even mean choosing them from an older one. In addition, combining the SNPs into a single instrument is another option if various studies report adequate variants. Where possible, it is best practice to choose SNPs found both in the discovery dataset and in a replication cohort, as these are likely to be more reliable.

In Table 1, we present the latest GWAS of sleep phenotypes (for a detailed list of SNPs reported in the GWAS see Supplementary Table 1). Of note is that some sleep traits are still lacking robust instruments. This is the case of sleep quality and multidimensional sleep (whereby rather than a series of single separate characteristics, sleep is thought of as a multidimensional construct with domains including regularity, satisfaction, alertness/sleepiness, timing, efficiency, and duration, among others) [77,78].

### 3 P-value

A common statistical approach, and usually the first step in MR studies, is to evaluate the level of statistical significance of the genetic variants associated with the exposure of interest and to include all variants at a given level of significance. The conventional threshold is p<5×10^−8^ [12]. This threshold is the equivalent of p<0.05 when corrected for the multiple testing based on performing a Bonferroni correction for all the independent common SNPs across the human genome and thus, it is referred to as “genome-wide level of significance”. Using this threshold has been shown to lead to robust results [79]. Nonetheless, given the more recent mega-GWAS because of access to large biobanks, there have been proposals to change it to p<5×10^−9^ to decrease the chances of false-positive associations. However, the latest MR studies of sleep traits have selected variants using the traditional threshold [30,44,51,52].

In the field of sleep, some of the first GWAS published discovered genetic variants for restless leg syndrome, a neurological disorder that causes involuntary leg movements during sleep [80,81]. Recently, a large GWAS reported that some of the variants previously associated with restless leg syndrome did not reach genome-wide significance, emphasising the need for stringent thresholds [82]. Even though it is important to consider the p-value threshold, this is not the only factor to consider when selecting variants for an MR study. The following sections will discuss other steps to assess whether variants are valid genetic instruments.

### 4 Linkage disequilibrium clumping

Linkage disequilibrium refers to the correlation between SNPs at different positions. This phenomenon occurs because of the physical proximity of variants on the chromosome [10]. In GWAS, the reported variants are often ‘clumped’ to near independence using distance-based or correlation-based thresholds [67].

The distance-based approach consists of pruning the variants to include those separated by a certain distance (usually 500.000 base pairs = 500 kilobases). In the correlation-based approach, only variants that are correlated at a certain threshold (usually r^2^<0.01, 0.1 or 0.2) are included [67]. Implementing the correlation-based approach, Broberg et al. (2021) [19], in their study about the association between insomnia and pain, decided to use an r^2^=0.6 as their primary threshold and an r^2^=0.1 as their secondary threshold. Cullel et al. (2021) [24] and Zhou et al. (2021) [42] clumped genetic variants considering both approaches, an r^2^<0.001 and a kb=10,000 distance, which is more conservative.

It is important to consider LD when selecting the variants as it could violate core MR assumptions. Genetic variants that are correlated with the variants used may have effects on competing risk factors. The LDkit (a graphical user interface software) or PLINK (open-source C/C++ toolset) could be used for calculating linkage disequilibrium [83,84]. Testing the association of potential confounders of the variants could reduce concerns about making invalid inferences due to LD [67].

### 5 Sample overlap

When selecting a GWA study, it is essential to understand in detail the sample studied. This is because when using genetic variants discovered in the analytical sample, a bias called “winner’s curse” may occur. This bias implies overestimating the strongest variant in the data under analysis [85]. An overestimation will generally occur when the associations with confounders are stronger than expected by chance. Thus, an overlap between the genetic variant discovery dataset and the data under analysis may overestimate the variant–outcome associations and lead to false-positive results [67]. To overcome this issue, Liu et al. (2022) [44] excluded data from participants in the UKB from their COVID-19 outcome dataset since their exposures (sleep and circadian phenotypes) were derived from this biobank.

The ideal situation to avoid this bias is having two non-overlapping datasets, what is called “Two-sample MR” [12]. MR-Base, a platform that integrates a database of GWAS results with an application programming interface, a web application and R packages, allows the automation of two-sample MR [86]. However, different datasets are not always available with the data or sample size necessary to perform the analysis. To mitigate potential issues with sample overlap, there are several alternatives thought to balance the risk of an imprecise estimation [67]. One option is to calculate the bias due to sample overlap, which can be done with the formulae from Burgess, Davies & Thompson (2016) [87]. Henry et al. (2019) [45] did this in their MR study about the impact of sleep duration on cognitive outcomes. In their study about the association of insomnia with depressive symptoms and subjective well-being, Zhou et al. (2021) [42] also calculated sample overlap finding a bias ranging from 3% to 14%.

Another possible solution is to perform the MR analysis using a reduced genetic instrument replicated in an independent cohort, which could be a good option as a sensitivity analysis for studies that are unable to bypass sample overlap. In our own MR study, which examined the association between genetically-instrumented habitual daytime napping (using 92 SNPs) and cognitive function and brain volume, we replicated our findings using a reduced instrument consisting of 17 SNPs that were replicated in an independent cohort (23andMe) with no sample overlap with UKB (our analytical sample) [88]. Additional analyses with this reduced instrument were largely consistent with our main findings. We are unaware of other studies using genetic instruments of sleep traits taking this approach. However, a study which investigated the relationship between glycaemia and cognitive function, brain structure and incident dementia, used a reduced genetic instrument for diabetes to avoid the “Winner’s curse” bias [89].

### 6 Type of exposure

When deciding which GWA study to select, it is important to prioritise well-measured/defined phenotypes used for identifying the genetic instruments. One aspect to consider is whether the phenotype was measured using self-reported data or an objective method (e.g. accelerometer-derived data). Many of the GWAS of sleep traits available are based on self-report questions, but some used and/or have been replicated with accelerometer-derived data, polysomnography or electronic medical records [69,90,91]. Moreover, those using self-reported data sometimes have support from objective measures. For example, Dashti et al. (2019) [92] tested whether the 78 loci found for self-reported habitual sleep duration (using a question on hours of sleep) in their GWA study were also associated with accelerometer-derived sleep estimates. Another study by Dashti et al. (2019) [27] found that the variants were also valid when sleep duration was determined by bed and wake times. Ideally, genetic instruments discovered and replicated based on objective data should be selected.

Moreover, it is essential to understand how the exposure was analysed. For example, Lane et al. (2019) [93] performed two parallel GWAS for frequent and any insomnia symptoms based on participants’ responses to the question “Do you have trouble falling asleep at night, or do you wake up in the middle of the night?”. For frequent insomnia, they considered participants who responded “usually” as cases and “never/rarely” as controls, with those reporting “sometimes” being excluded. For any insomnia, they considered participants who responded “sometimes” or “usually” as cases and “never/rarely” as controls. On the contrary, a GWA study by Jansen et al. (2019) [8], using the same question, defined insomnia cases as participants who answered “usually”, while participants who answered “never/rarely” or “sometimes” were defined as controls. In this example, insomnia symptoms are analysed in three different ways using the same underlying question. Understanding how the exposure was measured is crucial for adequately interpreting results.

Another crucial aspect of genetic instrument selection is whether the exposure is continuous or binary. It is well-established that continuous measures should be used where possible in MR [94]. However, using continuous exposures has the caveat that sometimes MR studies aim to test whether a particular disease status (e.g., insomnia) might be causally related to a specific outcome. Furthermore, some sleep traits are often considered binary: chronotype (evening vs. morning types), napping (frequent vs. infrequent nappers), and duration (longer vs. shorter sleepers), amongst others. In the case of using a binary exposure, it is important to be aware of its limitations. Burgess and Labrecque’s paper (2018) [94] explained that the problem arises when using a binary exposure that dichotomises a continuous variable (e.g. short/long sleep arises from dichotomised sleep duration). In the cases of MR studies using these types of exposures, the results should be conceptualised in terms of the underlying continuous risk factor.

### 7 Total (R^2^) and average strength (F-statistic) metrics

Selection of genetic instruments is often conducted by considering each variant’s effect size to avoid weak-instrument bias. This bias can occur when the genetic instruments explain a small proportion of the variance in the exposure. Weak instruments may lead to non-robust results and bias the estimates towards the confounded observational estimate [95].

Some of the most commonly used effect indicators are the proportion of the phenotypic variance explained by all of the genome-wide significant SNPs (R^2^) and the F-statistic obtained from regressing the exposure on the genetic instrument (in a multivariable linear regression) [62]. The R2 provides information about the total strength of the genetic variant, and usually, the larger, the better. Swerdlow et al. (2016) [62] argue that the R2 is the most useful effect metric when selecting genetic instruments for MR analysis. However, the F-statistic provides information about the average strength of the instrument, with an F>10 indicating that substantial weak instrument bias is unlikely [95].

Several options for obtaining F-statistics are available. If individual-level data are available for the exposure, the ‘Individual-level data regression’ approach can be performed. However, if individual-level data are not available and the R^2^ from the exposure GWA study is obtainable, the Cragg-Donald F-statistic method may be used [95]. This method uses the R^2^, sample size (n), and the number of instruments (k) to calculate the statistic (F=(n−k−1/k) (R^2^/1−R^2^)) [67]. Liu et al. (2021) [31] used this formula reporting a F-statistic of 143.24 in their study about the association between genetically-instrumented insomnia and cardiovascular diseases. When the R^2^ is unknown, the ‘t-statistic’ summary-level method can be used (F=ß^2^/SE^2^). In this case, the F-statistic will be an approximation because it uses the sample size for the discovery GWA study, not the one from the data under analysis. Finally, the “MendelianRandomization” R package allows the calculation of the F-statistic [13].

### 8 Number of SNPs

MR studies including large numbers of genetic variants are rapidly increasing. This growth is related to the proliferation of GWAS and the desire to obtain more precise estimates. However, as previously discussed, not all variants are valid IVs [96], and an enlarged set of genetic instruments is not always better [12]. Selecting a large number of variants could lead to a larger R^2^ but a weaker F-statistic and greater chances of including pleiotropic variants, violating a core MR assumption. Including more variants also allows the use of more robust methods, including common sensitivity analyses such as the MR-Egger test. On the contrary, fewer variants will lead to a lower R^2^ but potentially a greater F-statistic, which could lead to an instrument with insufficient power [96].

To understand how the strength of the instrument depends on the number of SNPs, we present Liu et al. (2022) [30] study on the relationship between sleep traits and glycated haemoglobin -HbA1c- (see Table 2). In this study, the F-statistic for all exposures was higher than 10 (which indicates an appropriate average strength), while the R^2^ ranged between 0,06 and 2,09%. In the case of long sleep duration, including fewer variants (five) lead to a low total strength (R^2^=0,06%) and a good average strength (F-statistic=41).

### 9 Minor allele frequency and palindromic variants

MAF is the proportion of minor alleles for a specific SNP in a given population [67]. In other words, it is the frequency at which the second most common allele occurs. Usually, GWAS identify common variants [97]; however, SNPs with a wide distribution of MAFs can sometimes be included. Some MR studies exclude variants with a low MAF because causal estimates from those variants may have low precision [96,98]. For example, Chen et al. (2021) (Chen et al., 2021) decided to remove variants with a MAF<1% in their study about the association between sleep traits and low bone mineral density. However, excluding variants with low MAF could mean removing variants associated with the exposure of interest. For example, low-frequency variants in *PERIOD3* have been associated with chronotype [7] and familial advanced sleep phase syndrome [99].

Another potential problem is palindromic variants because they can introduce ambiguity into the identification of the effect allele. A palindromic SNP occurs when the two possible alleles are complementary base pairs [86]. Additional care should be taken with palindromic variants because studies might report effects of the same SNP using different strands (e.g. a study reports an SNP with A/G alleles and another with T/C alleles). In those cases, the ambiguity can be identified if the effect allele frequency is reported and the MAF is substantially below 50% [100]. For example, if a specific SNP has alleles A/T, with allele A frequency being 0.11 in the GWA study and 0.91 in the data under study (both coding this allele as the effect allele) and both studies have the same ethnic origin, this means that authors used different reference strands. In this case, it is necessary to switch the direction of the effect in either the discovery GWA study or the analytical sample, a procedure called variant harmonisation [86].

However, if it is not possible to verify that alleles are correctly orientated, it may be necessary to take some precautions [67]. There are options to deal with this problem: replace the variants with suitable, non-palindromic linkage disequilibrium proxies, perform sensitivity analyses to evaluate the impact of these variants on the results or exclude them [100]. For example, in a study by Alimenti et al. (2021) [16] about causal links between habitual sleep duration/napping and macronutrient composition palindromic SNPs with MAF close to 0.50 were excluded and the remaining palindromic instruments were aligned based on their MAF.

### 10 Confounding

The third MR assumption states that the genetic variant-outcome association is unconfounded [101]. Violations of this assumption could be due to at least two different types of confounding. One is confounding by ancestry (e.g., if SNPs associated with sleep duration have higher/lower frequencies in different ancestry groups in the sample under study and additionally, cultural differences have an impact on the outcome under study), which could be controlled by restricting the sample to a single ancestry group, and/or adjusting for principal components of ancestry. A second source of confounding occurs if SNPs associated with the exposure of interest are also associated with common confounders of the relationship under study. One of the advantages of MR is that it exploits the fact that genotypes are not generally associated with confounders. However, such associations may occur, especially when using weak instruments or small samples [67]. Thus, it is important to test whether the genetic instruments are associated with confounders of the exposure outcome relationship [10].

To address this issue, authors must first identify common confounders previously reported between their exposures and outcomes. For example, in the case of the association between obstructive sleep apnea and hypertension, weight and age are proposed as two of the main confounding factors in this putative relationship [102]. For the long sleep-mortality association, some authors have argued that depression is most likely to confound this relationship [103]. Therefore, it is essential that, regardless of the exposure of interest, a literature review is carried out to identify the confounders to be considered.

Then, authors often statistically test associations between their genetic instrument and variables reported in the literature as potential confounders in the exposure-outcome association. This is crucial as MR aims to give causal estimates that are not biased due to confounding [67]. In the MR study performed by Henry et al. (2019) [45], the authors explored the validity of their instruments by testing associations of potential confounders (such as sex, age, educational attainment and use of sleep-inducing medication) with their sleep duration genetic score.

## Conclusions

In this article, we explored the criteria used for selecting genetic instruments for sleep traits in the context of MR, discussing how instrumental choice impacts analysis. We also presented GWAS of sleep phenotypes since 2016 and discussed MR studies using genetic sleep instruments to date. We are convinced that instrument selection is the most important decision when designing an MR study and that this is becoming even more important as the number of sleep genetic variants found in GWAS increases. We hope this review will aid researchers in designing robust MR studies and continue to elucidate our understanding of the causal role of sleep on health outcomes.

## Supplementary Material

Table S1

## Figures and Tables

**Table 1:** Genome-wide association studies of sleep phenotypes since 2016

Author (year^1^) [reference]	Phenotype	Phenotype measurement	N	Ancestry	Number of novel SNPs	Number of replicated SNPs from previous studies	Examples of health outcomes studied using these instruments
**Cade et al. (2016) [68]**	Obstructive sleep apnea	Apnea monitors and polysomnogra phy	12,558	Hispanic/La tino Americans	1 + 1 suggestive (P<5×10^−7^) (Apnea-hypopnea index) / 1 + 4 suggestive (Respiratory event duration) / 2 suggestive (Sleep Sp O2)	-	Cancer [28]
**Hu et al. (2016) [104]**	Morningness	Sel-freported questions	89,283	European	15	-	Inflammatory bowel disease [22]
**Jones et al. (2016) [105]**	Morningness and sleep duration	Self-reported question	128,266	European (validation in Koreans)	10 (morningness) + 2 suggestive (P<1x10-4) / 3 (sleep duration)	3 + 1 suggestive from Lane et al. (2016) (morningness)	Caffeine consumption [60]
**Lane et al. (2016) [106]**	Chronotype	Self-reported question	100,420	European	12	-	-
**Hammersch ag et al. (2017) [75]**	**l** Insomnia symptoms	Self-reported question validated with questionnaires and a structured interview	113,006	European	11 + 2 suggestive (P<2×10^−3^)	1 + 1 suggestive from Lane et al. (2017)	Caffeine consumption [60]; Peptic ulcer disease [40]
**Lane et al. (2017) [107]**	Sleep duration, insomnia symptoms, excessive daytime sleepiness & composite sleep trait^2^	Self-reported questions	112,586	European	1 + 3 suggestive (P<5×10^−7^) (sleep duration) / 5 + 3 suggestive (insomnia symptoms) / 3 + 7 suggestive (excessive daytime sleepiness) / 3 (composite sleep trait)	1 suggestive from Jones et al. (2016) (sleep duration) / 3 from Jones et al. (2016) & Lane et al. (2016) (composite sleep trait)	-
**Schormair et al. (2017) [108]**	Restless legs syndrome	Interviews and self-reported question	110,851	European	19	1 from Lane et al. (2017)	Parkinson’s disease [53]; Essential tremor [109]
**Chen et al. (2018)[69]**	Obstructive sleep apnea	Polysomnogra phy	19,733	Multiancestry	23 suggestive (P<1.0×10^−6^) (Apnea-Hypopnea Index total) / 10 suggestive (Apnea-Hypopnea Index-Non-Rapid Eye Movement) / 7 suggestive (Apnea-Hypopnea Index-Rapid Eye Movement).	1 suggestive from Cade et al. (2016) (Apnea-Hypopnea Index total)	Amyotrophic lateral sclerosis [57]; Neurodegenerative diseases [24]; Parkinson’s disease [57]
**Doherty et al. (2018) [91]**	Sleep duration	Accelerometer data	91,105	European	7	1 from Lane et al. (2017)	-
**Ferguson et al. (2018) [110]**	Low relative amplitude	Accelerometer data	71,500	European	3	‘	-
**Stein et al. (2018)[73]**	Insomnia disorder	Questionnaire	17,651	Multiancestry	4 + 8 suggestive (P<1×10^−6^)	-	-
**Dashti et al. (2019) [6]**	Sleep duration	Self-reported question supported by accelerometer data	446,118	European	77	1 from Jones et al. (2019)	Alzheimer’s disease [17,111]; Amyotrophic lateral sclerosis [57]; Atrial fibrillation [41]; Cancer [59,112]; Cardiovascular diseases [15]; COVID-19 [44]; Dietary intake [16]; Fracture [58]; Glycated hemoglobin [30]; Heart failure [41]; Heel bone mineral density [20]; Intracranial aneurysm and Aneurysmal subarachnoid hemorrhage [55]; Ischemic stroke [113]; Longevity [50]; Major depressive disorder [111]; Metabolomic traits [18]; Migraine [114]; Osteoarthritis [33]; Parkinson’s disease [57]; Periodontal disease [43]; Renal function [32]; Stroke [37]
**Jansen et al. (2019) [8]**	Insomnia symptoms	Self-reported question	1,331,0 10	European	243	5 from Jones et al. (2016, 2019), Lane et al. (2017) & Doherty et al. (2018)	Asthma [29]; Body composition [52]; Cancer [35,51]; Cardiovascular conditions [31,39]; Depressive symptoms [42]; Fracture [58]; Hypertension [115]; Intracranial aneurysm and Aneurysmal subarachnoid hemorrhage [55]; Longevity [50]; Major depressive disorder [116]; Metabolomic traits [18]; Migraine [23]; Osteoarthritis [33]; Pain diagnoses [19]; Subjective well-being [42]; Suicidal behavior [46]
**Jones et al. (2019) [7]**	Morningness	Self-reported question	697,828	European	344	7 from Hu et al. (2016), Lane et al. (2016, 2017) & Jones et al. (2016)	Alzheimer’s disease [17,111]; Amyotrophic lateral sclerosis [57]; Cancer [36,51,112]; COVID-19 [44]; Depressive symtoms [34]; Food intake [26]; Fracture [58]; General anxiety disorder [34]; General wellbeing [34]; Glycated hemoglobin [30]; Heel bone mineral density [20]; Inflammatory bowel disease [22]; Ischemic stroke [113]; Major depressive disorder [25,34,111]; Metabolomic traits [18]; Migraine [114]; Neurodegenerative diseases [24]; Parkinson’s disease [57]
**Jones et al. (2019) [117]**	Sleep traits derived by acceleromete r data	Accelerometer data	85,670	European	9 (sleep duration) / 1 (sleep midpoint) / 4 (sleep eficiency) / 20 (number of sleep episodes).	2 from Lane et al. (2017) & Doherty et al. (2018) (sleep duration) / 1 from Lane et al. (2017) (sleep eficiency) / 1 from Jansen et al. (2019) (number of sleep episodes).	Alzheimer’s disease [17,111]; Amyotrophic lateral sclerosis [57]; Cancer [36]; Major depressive disorder [111]; Neurodegenerative diseases [24]; Parkinson’s disease [57]
**Lane et al. (2019) [93]**	Insomnia symptoms	Self-reported question	453,379	European	51	6 from Lane et al. (2017), Doherty et al. (2018)& Jansen et al. (2019)	Alzheimer’s disease [17,111]; Amyotrophic lateral sclerosis [57]; Cancer [112]; COVID-19 [44]; Heel bone mineral density [20]; Inflammatory bowel disease [22]; Ischemic stroke [113]; Major depressive disorder [111]; Migraine [114]; Neurodegenerative diseases [24]; Pain diagnoses [19]; Parkinson’s disease [57]
**Wang et al. (2019) [118]**	Daytime sleepiness	Self-reported question	452,071	European	40	2 from Jones et al. (2016) & Jansen et al. (2019)	Alzheimer’s disease [17]; Amyotrophic lateral sclerosis [57]; COVID-19 [44]; Glycated hemoglobin [30]; Heel bone mineral density [20]; Inflammatory bowel disease [22]; Migraine [114]; Neurodegenerative diseases [24]; Parkinson’s disease [57]
**Campos et al. (2020) [119]**	Snoring	Self-reported question	408,000	European	41	-	Amyotrophic lateral sclerosis [57]; Atrial fibrillation [54]; Body mass index [54]; Fracture [58]; Major depressive disorder [56]; Parkinson’s disease [57]
**Didriksen et al. (2020) [120]**	Restless legs syndrome	Clinical diagnosis and questionnaire	480,982	European	3 + 2 suggestive at (P<7×10^−7^)	20 from Lane et al. (2017) & Schormair et al. (2017)	-
**Farias Tempaku et al. (2020) [70]**	Obstructive sleep apnea	Polysomnogra phy	1074	Multiancestry	2 + 21 suggestive (P<5×10^−6^)	-	-
**Song et al. (2020) [90]**	Insomnia disorder	Electronic health records	18,055	European	1	-	-
**Dashti et al. (2021) [121]**	Daytime napping	Self-reported question	452,633	European	119	4 from Jones et al. (2016, 2019) & Jansen et al. (2019)	COVID-19** [44];** Dietary intake [16]; Glycated hemoglobin [30]; Inflammatory bowel disease [22]; Migraine [114]
**Khoury et al. (2021)** [71]	Sleep quality	Questionnaire	2868	Multiancestry	3 + 11 suggestive (P≤5×10^−7^)	-	-
**Strausz et al. (2021) [122]**	Obstructive sleep apnea	Electronic health records	217,955	European	5	-	Atrial fibrillation [21]
**Yao et al. (2022) [123]**	Sleep health score^3^	Self-reported questions	336,463	European	31	1 from Lane et al. (2017)	-
**Watanabe et al. (2022) [65]**	Insomnia symptoms	Self-reported questions	2,365,0 10	European	364	190 from Jansen et al. (2019), Lane et al. (2017, 2019) & Hammerschlag et al. (2017)	Sepsis [124]
**Austin-Zimmerman et al. (2023) [74]**	Sleep duration	Self-reported questions	493,142	Multi-ancestry	71 (short sleep duration)	13 (short sleep duration) + 1 (long sleep duration) from Gottlieb et al. (2015) & Song et al. (2020)	-

1 We reported studies since 2016 because at that time there was a proliferation of GWAS of sleep phenotypes (previously most studies were done on Restless legs syndrome).

2 Composite trait of sleep duration, insomnia symptoms, excessive daytime sleepiness and chronotype.

3 Overall assessment of sleep duration, snoring, insomnia symptoms, chronotype, and daytime dozing.

**Table 2:** Instrument strength metrics from Liu et al. (2022)

Information of the discovery GWAS						Two-sample MR		
Author (year) [reference]	Trait	n	Cohort	nSNPs identified	R^2^	nSNPs merged	R^2^*	F-statistics**
Jansen et al. (2019) [8]	Insomnia symptoms	1.331.010	UKB/23andMe	248	2,60%	179	0,55%	41
Dashti et al. (2019) [92]	Sleep duration	446.118	UKB	78	0,69%	54	0,49%	41
Dashti et al. (2019) [92]	Short sleep duration	411.934	UKB	27	-	20	0,13%	27
Dashti et al. (2019) [92]	Long sleep duration	339.926	UKB	8	-	5	0,06%	41
Wang et al. (2019) [118]	Daytime sleepiness	452.071	UKB	37	-	26	0,25%	44
Dashti et al. (2021) [121]	Daytime napping	993.966	UKB/23andMe	108	1,10%	71	0,56%	79
Jones et al. (2019) [7]	Chronotype	697.828	UKB/23andMe	351	-	250	2,09%	60

GWAS: Genome-wide association studies; nSNPs: number of single-nucleotide polymorphism; UKB: UK Biobank.

* R^2^ was calculated via Sum(R2i==K*Fi/(N--K--1+K*Fi), K=1, Fi=BetaXGi^2/seBetaXGi^2 (BetaXGi and seBetaXGi were obtained from the discovery GWAS).

** F statistic was calculated via Cragg-Donald method.

## References

[1] Borbely AA (1982). A Two Process Model of Sleep Regulation.

[2] Chaput J-P, Shiau J (2019). Routinely assessing patients’ sleep health is time well spent. Prev Med Rep.

[3] Kalmbach DA, Schneider LD, Cheung J, Bertrand SJ, Kariharan T, Pack AI (2017). Genetic Basis of Chronotype in Humans: Insights From Three Landmark GWAS. Sleep.

[4] Kocevska D, Barclay NL, Bramer WM, Gehrman PR, Van Someren EJW (2021). Heritability of sleep duration and quality: A systematic review and meta-analysis. Sleep Med Rev.

[5] Garfield V (2021). Sleep duration: A review of genome-wide association studies (GWAS) in adults from 2007 to 2020. Sleep Med Rev.

[6] Dashti HS, Jones SE, Wood AR, Lane JM, Van Hees VT, Wang H (2019). Genome-wide association study identifies genetic loci for self-reported habitual sleep duration supported by accelerometer-derived estimates. Nat Commun.

[7] Jones SE, Lane JM, Wood AR, van Hees VT, Tyrrell J, Beaumont RN (2019). Genome-wide association analyses of chronotype in 697,828 individuals provides insights into circadian rhythms. Nat Commun.

[8] Jansen PR, Watanabe K, Stringer S, Skene N, Bryois J, Hammerschlag AR (2019). Genome-wide analysis of insomnia in 1,331,010 individuals identifies new risk loci and functional pathways. Nat Genet.

[9] Medic G, Wille M, Hemels M (2017). Short- and long-term health consequences of sleep disruption. Nat Sci Sleep.

[10] Smith GD, Ebrahim S (2003). ‘Mendelian randomization’: can genetic epidemiology contribute to understanding environmental determinants of disease?. Int J Epidemiol.

[11] Evans DM, Davey Smith G (2015). Mendelian Randomization: New Applications in the Coming Age of Hypothesis-Free Causality. Annu Rev Genomics Hum Genet.

[12] Burgess S, Davey Smith G, Davies NM, Dudbridge F, Gill D, Glymour MM (2020). Guidelines for performing Mendelian randomization investigations. Wellcome Open Res.

[13] Yavorska OO, Burgess S (2017). MendelianRandomization: an R package for performing Mendelian randomization analyses using summarized data. Int J Epidemiol.

[14] Spiller W, Davies NM, Palmer TM (2019). Software application profile: mrrobust — a tool for performing two-sample summary Mendelian randomization analyses. Int J Epidemiol.

[15] Ai S, Zhang J, Zhao G, Wang N, Li G, So H-C (2021). Causal associations of short and long sleep durations with 12 cardiovascular diseases: linear and nonlinear Mendelian randomization analyses in UK Biobank. Eur Heart J.

[16] Alimenti K, Chen A, Saxena R, Dashti HS (2021). Habitual Sleep Duration, Daytime Napping, and Dietary Intake: A Mendelian Randomization Study. Curr Dev Nutr.

[17] Anderson EL, Richmond RC, Jones SE, Hemani G, Wade KH, Dashti HS (2021). Is disrupted sleep a risk factor for Alzheimer’s disease? Evidence from a two-sample Mendelian randomization analysis. Int J Epidemiol.

[18] Bos MM, Goulding NJ, Lee MA, Hofman A, Bot M, Pool R (2021). Investigating the relationships between unfavourable habitual sleep and metabolomic traits: evidence from multi-cohort multivariable regression and Mendelian randomization analyses. BMC Med.

[19] Broberg M, Karjalainen J, FinnGen, Ollila HM (2021). Mendelian randomization highlights insomnia as a risk factor for pain diagnoses. Sleep.

[20] Chen J, Zhang J, So HC, Ai S, Wang N, Tan X (2021). Association of Sleep Traits and Heel Bone Mineral Density: Observational and Mendelian Randomization Studies. J Bone Miner Res.

[21] Chen L, Sun X, He Y, Lu Y, Zheng L (2022). Obstructive sleep apnea and atrial fibrillation: insights from a bidirectional Mendelian randomization study. BMC Med Genomics.

[22] Chen M, Peng W-Y, Tang T-C, Zheng H (2021). Differential Sleep Traits Have No Causal Effect on Inflammatory Bowel Diseases: A Mendelian Randomization Study. Front Pharmacol.

[23] Chu S, Wu Z, Wu Z, Wu J, Qian Y (2021). Association Between Insomnia and Migraine Risk: A Case–Control and Bidirectional Mendelian Randomization Study. Pharmacogenomics Pers Med.

[24] Cullell N, Cárcel-Márquez J, Gallego-Fábrega C, Muiño E, Llucià-Carol L, Lledós M (2021). Sleep/wake cycle alterations as a cause of neurodegenerative diseases: A Mendelian randomization study. Neurobiol Aging.

[25] Daghlas I, Lane JM, Saxena R, Vetter C (2021). Genetically Proxied Diurnal Preference, Sleep Timing, and Risk of Major Depressive Disorder. JAMA Psychiatry.

[26] Dashti HS, Chen A, Daghlas I, Saxena R (2020). Morning diurnal preference and food intake: a Mendelian randomization study. Am J Clin Nutr.

[27] Dashti HS, Redline S, Saxena R (2019). Polygenic risk score identifies associations between sleep duration and diseases determined from an electronic medical record biobank. Sleep.

[28] Gao X-L, Jia Z-M, Zhao F-F, An D-D, Wang B, Cheng E-J (2020). Obstructive sleep apnea syndrome and causal relationship with female breast cancer: a mendelian randomization study. Aging.

[29] Kim DJ, Ha T-W, Jung HU, Baek EJ, Lee WJ, Kim HK (2021). Characterisation of insomnia as an environmental risk factor for asthma via Mendelian randomization and gene environment interaction. Sci Rep.

[30] Liu J, Richmond RC, Bowden J, Barry C, Dashti HS, Daghlas I (2022). Assessing the Causal Role of Sleep Traits on Glycated Hemoglobin: A Mendelian Randomization Study. Diabetes Care.

[31] Liu X, Li C, Sun X, Yu Y, Si S, Hou L (2021). Genetically Predicted Insomnia in Relation to 14 Cardiovascular Conditions and 17 Cardiometabolic Risk Factors: A Mendelian Randomization Study. J Am Heart Assoc.

[32] Mazidi M, Shekoohi N, Katsiki N, Banach M (2021). Longer sleep duration may negatively affect renal function. Int Urol Nephrol.

[33] Ni J, Zhou W, Cen H, Chen G, Huang J, Yin K (2022). Evidence for causal effects of sleep disturbances on risk for osteoarthritis: a univariable and multivariable Mendelian randomization study. Osteoarthritis Cartilage.

[34] O’Loughlin J, Casanova F, Jones SE, Hagenaars SP, Beaumont RN, Freathy RM (2021). Using Mendelian Randomisation methods to understand whether diurnal preference is causally related to mental health. Mol Psychiatry.

[35] Shen J, Zhou H, Liu J, Zhang Y, Zhou T, Chen G (2021). Genetic Liability to Insomnia and Lung Cancer Risk: A Mendelian Randomization Analysis. Front Genet.

[36] Sun X, Ye D, Jiang M, Qian Y, Mao Y (2021). Genetically proxied morning chronotype was associated with a reduced risk of prostate cancer. Sleep.

[37] Titova OE, Yuan S, Baron JA, Lindberg E, Michaëlsson K, Larsson SC (2022). Sleep-disordered breathing-related symptoms and risk of stroke: cohort study and Mendelian randomization analysis. J Neurol.

[38] Yu M, Du Y, Liu K, Liang X, Huang C, He R (2021). Sleep duration and auditory hallucinations: Genetic correlation and two-sample Mendelian randomization study. J Affect Disord.

[39] Yuan S, Mason AM, Burgess S, Larsson SC (2021). Genetic liability to insomnia in relation to cardiovascular diseases: a Mendelian randomisation study. Eur J Epidemiol.

[40] Zha L-F, Dong J-T, Wang J-L, Chen Q-W, Wu J-F, Zhou Y-C (2021). Effects of Insomnia on Peptic Ulcer Disease Using Mendelian Randomization. Oxid Med Cell Longev.

[41] Zhao J, Yang F, Zhuo C, Wang Q, Qu Z, Wang Q (2021). Association of Sleep Duration With Atrial Fibrillation and Heart Failure: A Mendelian Randomization Analysis. Front Genet.

[42] Zhou F, Guo Y, Wang Z, Liu S, Xu H (2021). Assessing the causal associations of insomnia with depressive symptoms and subjective well-being: a bidirectional Mendelian randomization study. Sleep Med.

[43] Zhou F, Liu Z, Guo Y, Xu H (2021). Association of short sleep with risk of periodontal disease: A meta-analysis and Mendelian randomization study. J Clin Periodontol.

[44] Liu Z, Luo Y, Su Y, Wei Z, Li R, He L (2022). Associations of sleep and circadian phenotypes with COVID-19 susceptibility and hospitalization: an observational cohort study based on the UK Biobank and a two-sample Mendelian randomization study. Sleep.

[45] Henry A, Katsoulis M, Masi S, Fatemifar G, Denaxas S, Acosta D (2019). The relationship between sleep duration, cognition and dementia: a Mendelian randomization study. Int J Epidemiol.

[46] Nassan M, Daghlas I, Winkelman JW, Dashti HS, Saxena R, International Suicide Genetics Consortium (2022). Genetic evidence for a potential causal relationship between insomnia symptoms and suicidal behavior: a Mendelian randomization study. Neuropsychopharmacology.

[47] Paz V, Dashti HS, Garfield V (2022). Is there an association between daytime napping, cognitive function and brain volume? A Mendelian randomisation study in the UK Biobank. Prepr MedRxiv.

[48] Baranova A, Cao H, Zhang F (2022). Shared genetic liability and causal effects between major depressive disorder and insomnia. Hum Mol Genet.

[49] van Oort S, Beulens JWJ, van Ballegooijen AJ, Handoko ML, Larsson SC (2020). Modifiable lifestyle factors and heart failure: A Mendelian randomization study. Am Heart J.

[50] van Oort S, Beulens JWJ, Ballegooijen AJ, Burgess S, Larsson SC (2021). Cardiovascular risk factors and lifestyle behaviours in relation to longevity: a Mendelian randomization study. J Intern Med.

[51] Chen F, Wen W, Long J, Shu X, Yang Y, Shu X (2022). Mendelian randomization analyses of 23 known and suspected risk factors and biomarkers for breast cancer overall and by molecular subtypes. Int J Cancer.

[52] Chen Y, Li C, Cheng S, Pan C, Zhang H, Zhang J (2022). The Causal Relationships Between Sleep-related Phenotypes and Body Composition: A Mendelian Randomized Study. J Clin Endocrinol Metab.

[53] Estiar MA, Senkevich K, Yu E, Varghaei P, Krohn L, Bandres-Ciga S (2021). Lack of Causal Effects or Genetic Correlation between Restless Legs Syndrome and Parkinson’s Disease. Mov Disord.

[54] Ardissino M, Reddy RK, Slob EAW, Patel KHK, Ryan DK, Gill D (2022). Sleep Disordered Breathing, Obesity and Atrial Fibrillation: A Mendelian Randomisation Study. Genes.

[55] Karhunen V, Bakker MK, Ruigrok YM, Gill D, Larsson SC (2021). Modifiable Risk Factors for Intracranial Aneurysm and Aneurysmal Subarachnoid Hemorrhage: A Mendelian Randomization Study. J Am Heart Assoc.

[56] Chen G, Xie J, Liu W, Liang T, Liao X, Liao W (2022). Association between depression and sleep apnoea: a Mendelian randomisation study. ERJ Open Res.

[57] Di H, Zhu Y, Xia W, Meng X, Zhang M, Xu M (2022). Bidirectional Mendelian randomization to explore the causal relationships between Sleep traits, Parkinson’s disease and Amyotrophic lateral sclerosis. Sleep Med.

[58] Qian Y, Xia J, Liu K-Q, Xu L, Xie S-Y, Chen G-B (2021). Observational and genetic evidence highlight the association of human sleep behaviors with the incidence of fracture. Commun Biol.

[59] Titova OE, Michaëlsson K, Vithayathil M, Mason AM, Kar S, Burgess S (2021). Sleep duration and risk of overall and 22 site-specific cancers: A Mendelian randomization study. Int J Cancer.

[60] Treur JL, Gibson M, Taylor AE, Rogers PJ, Munafò MR (2018). Investigating genetic correlations and causal effects between caffeine consumption and sleep behaviours. J Sleep Res.

[61] Haycock PC, Burgess S, Wade KH, Bowden J, Relton C, Davey Smith G (2016). Best (but oft-forgotten) practices: the design, analysis, and interpretation of Mendelian randomization studies. Am J Clin Nutr.

[62] Swerdlow DI, Kuchenbaecker KB, Shah S, Sofat R, Holmes MV, White J (2016). Selecting instruments for Mendelian randomization in the wake of genome-wide association studies. Int J Epidemiol.

[63] Brion M-JA, Shakhbazov K, Visscher PM (2013). Calculating statistical power in Mendelian randomization studies. Int J Epidemiol.

[64] Dauvilliers Y, Maret S, Tafti M (2005). Genetics of normal and pathological sleep in humans. Sleep Med Rev.

[65] Watanabe K, Jansen PR, Savage JE, Nandakumar P, Wang X, 23andMe Research Team (2022). Genome-wide meta-analysis of insomnia prioritizes genes associated with metabolic and psychiatric pathways. Nat Genet.

[66] Lane JM, Qian J, Mignot E, Redline S, Scheer FAJL, Saxena R (2023). Genetics of circadian rhythms and sleep in human health and disease. Nat Rev Genet.

[67] Burgess S, Thompson SG (2021). Mendelian Randomization: Methods for Causal Inference Using Genetic Variants. CRC Press.

[68] Cade BE, Chen H, Stilp AM, Gleason KJ, Sofer T, Ancoli-Israel S (2016). Genetic Associations with Obstructive Sleep Apnea Traits in Hispanic/Latino Americans. Am J Respir Crit Care Med.

[69] Chen H, Cade BE, Gleason KJ, Bjonnes AC, Stilp AM, Sofer T (2018). Multiethnic Meta-Analysis Identifies *RAI1* as a Possible Obstructive Sleep Apnea–related Quantitative Trait Locus in Men. Am J Respir Cell Mol Biol.

[70] Farias Tempaku P, Leite Santoro M, Bittencourt L, D’Almeida V, Iole Belangero S, Tufik S (2020). Genome-wide association study reveals two novel risk alleles for incident obstructive sleep apnea in the EPISONO cohort. Sleep Med.

[71] Khoury S, Wang Q-P, Parisien M, Gris P, Bortsov AV, Linnstaedt SD (2021). Multi-ethnic GWAS and meta-analysis of sleep quality identify MPP6 as a novel gene that functions in sleep center neurons. Sleep.

[72] Scammell BH, Tchio C, Song Y, Nishiyama T, Louie TL, Dashti HS (2023). Multi-ancestry genome-wide analysis identifies shared genetic effects and common genetic variants for self-reported sleep duration. Hum Mol Genet.

[73] Stein MB, McCarthy MJ, Chen C-Y, Jain S, Gelernter J, He F (2018). Genome-wide analysis of insomnia disorder. Mol Psychiatry.

[74] Austin-Zimmerman I, Levey DF, Giannakopoulou O, Deak JD, Galimberti M, Adhikari K (2023). Genome-wide association studies and cross-population meta-analyses investigating short and long sleep duration. Nat Commun.

[75] Hammerschlag AR, Stringer S, de Leeuw CA, Sniekers S, Taskesen E, Watanabe K (2017). Genome-wide association analysis of insomnia complaints identifies risk genes and genetic overlap with psychiatric and metabolic traits. Nat Genet.

[76] Mong JA, Cusmano DM (2016). Sex differences in sleep: impact of biological sex and sex steroids. Philos Trans R Soc B Biol Sci.

[77] Buysse DJ (2014). Sleep Health: Can We Define It? Does It Matter?. Sleep.

[78] Wallace ML, Buysse DJ, Redline S, Stone KL, Ensrud K, Leng Y (2019). Multidimensional Sleep and Mortality in Older Adults: A Machine-Learning Comparison With Other Risk Factors. J Gerontol Ser A.

[79] Chen Z, Boehnke M, Wen X, Mukherjee B (2021). Revisiting the genome-wide significance threshold for common variant GWAS.

[80] Stefansson H, Rye DB, Hicks A, Petursson H, Ingason A, Thorgeirsson TE (2007). A Genetic Risk Factor for Periodic Limb Movements in Sleep. N Engl J Med.

[81] Winkelmann J, Schormair B, Lichtner P, Ripke S, Xiong L, Jalilzadeh S (2007). Genome-wide association study of restless legs syndrome identifies common variants in three genomic regions. Nat Genet.

[82] Schormair B, Zhao C, Salminen AV, Oexle K, Winkelmann J, International EU-RLS-GENE Consortium (2022). Reassessment of candidate gene studies for idiopathic restless legs syndrome in a large genome-wide association study dataset of European ancestry. Sleep.

[83] Chang CC, Chow CC, Tellier LC, Vattikuti S, Purcell SM, Lee JJ (2015). Second-generation PLINK: rising to the challenge of larger and richer datasets. GigaScience.

[84] Tang Y, Li Z, Wang C, Liu Y, Yu H, Wang A (2020). LDkit: a parallel computing toolkit for linkage disequilibrium analysis. BMC Bioinformatics.

[85] Taylor AE, Davies NM, Ware JJ, VanderWeele T, Smith GD, Munafò MR (2014). Mendelian randomization in health research: Using appropriate genetic variants and avoiding biased estimates. Econ Hum Biol.

[86] Hemani G, Zheng J, Elsworth B, Wade KH, Haberland V, Baird D (2018). The MR-Base platform supports systematic causal inference across the human phenome. eLife.

[87] Burgess S, Davies NM, Thompson SG (2016). Bias due to participant overlap in two-sample Mendelian randomization. Genet Epidemiol.

[88] Paz V, Dashti HS, Garfield V (2023). Is there an association between daytime napping, cognitive function, and brain volume? A Mendelian randomization study in the UK Biobank. Sleep Health.

[89] Garfield V, Farmaki A-E, Fatemifar G, Eastwood SV, Mathur R, Rentsch CT (2021). Relationship Between Glycemia and Cognitive Function, Structural Brain Outcomes, and Dementia: A Mendelian Randomization Study in the UK Biobank. Diabetes.

[90] Song W, Torous J, Kossowsky J, Chen C-Y, Huang H, Wright A (2020). Genome-wide association analysis of insomnia using data from Partners Biobank. Sci Rep.

[91] Doherty A, Smith-Byrne K, Ferreira T, Holmes MV, Holmes C, Pulit SL (2018). GWAS identifies 14 loci for device-measured physical activity and sleep duration. Nat Commun.

[92] Dashti HS, Jones SE, Wood AR, Lane JM, van Hees VT, Wang H (2019). Genome-wide association study identifies genetic loci for self-reported habitual sleep duration supported by accelerometer-derived estimates. Nat Commun.

[93] Lane JM, Jones SE, Dashti HS, Wood AR, Aragam KG, HUNT All In Sleep (2019). Biological and clinical insights from genetics of insomnia symptoms. Nat Genet.

[94] Burgess S, Labrecque JA (2018). Mendelian randomization with a binary exposure variable: interpretation and presentation of causal estimates. Eur J Epidemiol.

[95] Burgess S, Thompson SG, CRP CHD Genetics Collaboration (2011). Avoiding bias from weak instruments in Mendelian randomization studies. Int J Epidemiol.

[96] Bowden J, Davey Smith G, Burgess S (2015). Mendelian randomization with invalid instruments: effect estimation and bias detection through Egger regression. Int J Epidemiol.

[97] Dehghan A, Evangelou E (2018). Genet Epidemiol.

[98] Tabangin ME, Woo JG, Martin LJ (2009). The effect of minor allele frequency on the likelihood of obtaining false positives. BMC Proc.

[99] Zhang L, Hirano A, Hsu P-K, Jones CR, Sakai N, Okuro M (2016). A *PERIOD3* variant causes a circadian phenotype and is associated with a seasonal mood trait. Proc Natl Acad Sci.

[100] Hartwig FP, Davies NM, Hemani G, Davey Smith G (2016). Two-sample Mendelian randomization: avoiding the downsides of a powerful, widely applicable but potentially fallible technique. Int J Epidemiol.

[101] Davies NM, Holmes MV, Davey Smith G (2018). Reading Mendelian randomisation studies: a guide, glossary, and checklist for clinicians. BMJ.

[102] Feng J, Chen B-Y (2009). Prevalence and incidence of hypertension in obstructive sleep apnea patients and the relationship between obstructive sleep apnea and its confounders. Chin Med J (Engl).

[103] Grandner MA, Drummond SPA (2007). Who are the long sleepers? Towards an understanding of the mortality relationship. Sleep Med Rev.

[104] Hu Y, Shmygelska A, Tran D, Eriksson N, Tung JY, Hinds DA (2016). GWAS of 89,283 individuals identifies genetic variants associated with self-reporting of being a morning person. Nat Commun.

[105] Jones SE, Tyrrell J, Wood AR, Beaumont RN, Ruth KS, Tuke MA (2016). Genome-Wide Association Analyses in 128,266 Individuals Identifies New Morningness and Sleep Duration Loci. PLOS Genet.

[106] Lane JM, Vlasac I, Anderson SG, Kyle SD, Dixon WG, Bechtold DA (2016). Genome-wide association analysis identifies novel loci for chronotype in 100,420 individuals from the UK Biobank. Nat Commun.

[107] Lane JM, Liang J, Vlasac I, Anderson SG, Bechtold DA, Bowden J (2017). Genome-wide association analyses of sleep disturbance traits identify new loci and highlight shared genetics with neuropsychiatric and metabolic traits. Nat Genet.

[108] Schormair B, Zhao C, Bell S, Tilch E, Salminen AV, Pütz B (2017). Identification of novel risk loci for restless legs syndrome in genome-wide association studies in individuals of European ancestry: a meta-analysis. Lancet Neurol.

[109] Liao C, Houle G, He Q, Laporte AD, Girard SL, Dion PA (2019). Investigating the association and causal relationship between restless legs syndrome and essential tremor. Parkinsonism Relat Disord.

[110] Ferguson A, Lyall LM, Ward J, Strawbridge RJ, Cullen B, Graham N (2018). Genome-Wide Association Study of Circadian Rhythmicity in 71,500 UK Biobank Participants and Polygenic Association with Mood Instability. EBioMedicine.

[111] Huang J, Zuber V, Matthews PM, Elliott P, Tzoulaki J, Dehghan A (2020). Sleep, major depressive disorder, and Alzheimer disease: A Mendelian randomization study. Neurology.

[112] Richmond RC, Anderson EL, Dashti HS, Jones SE, Lane JM, Strand LB (2019). Investigating causal relations between sleep traits and risk of breast cancer in women: mendelian randomisation study. BMJ.

[113] Cai H, Liang J, Liu Z, Fang L, Zheng J, Xu J (2020). Causal Effects of Sleep Traits on Ischemic Stroke and Its Subtypes: A Mendelian Randomization Study. Nat Sci Sleep.

[114] Daghlas I, Vgontzas A, Guo Y, Chasman DI, Saxena R, International Headache Genetics Consortium (2020). Habitual sleep disturbances and migraine: a Mendelian randomization study. Ann Clin Transl Neurol.

[115] van Oort S, Beulens JWJ, van Ballegooijen AJ, Grobbee DE, Larsson SC (2020). Association of Cardiovascular Risk Factors and Lifestyle Behaviors With Hypertension: A Mendelian Randomization Study. Hypertension.

[116] Cai L, Bao Y, Fu X, Cao H, Baranova A, Zhang X (2021). Causal links between major depressive disorder and insomnia: A Mendelian randomisation study. Gene.

[117] Jones SE, van Hees VT, Mazzotti DR, Marques-Vidal P, Sabia S, van der Spek A (2019). Genetic studies of accelerometer-based sleep measures yield new insights into human sleep behaviour. Nat Commun.

[118] Wang H, Lane JM, Jones SE, Dashti HS, Ollila HM, Wood AR (2019). Genome-wide association analysis of self-reported daytime sleepiness identifies 42 loci that suggest biological subtypes. Nat Commun.

[119] Campos AI, García-Marín LM, Byrne EM, Martin NG, Cuéllar-Partida G, Rentería ME (2020). Insights into the aetiology of snoring from observational and genetic investigations in the UK Biobank. Nat Commun.

[120] Didriksen M, Nawaz MS, Dowsett J, Bell S, Erikstrup C, Pedersen OB (2020). Large genome-wide association study identifies three novel risk variants for restless legs syndrome. Commun Biol.

[121] Dashti HS, Daghlas I, Lane JM, Huang Y, Udler MS, Wang H (2021). Genetic determinants of daytime napping and effects on cardiometabolic health. Nat Commun.

[122] Strausz S, Ruotsalainen S, Ollila HM, Karjalainen J, Kiiskinen T, Reeve M (2021). Genetic analysis of obstructive sleep apnoea discovers a strong association with cardiometabolic health. Eur Respir J.

[123] Yao Y, Jia Y, Wen Y, Cheng B, Cheng S, Liu L (2022). Genome-Wide Association Study and Genetic Correlation Scan Provide Insights into Its Genetic Architecture of Sleep Health Score in the UK Biobank Cohort. Nat Sci Sleep.

[124] Thorkildsen MS, Gustad LT, Mohus RM, Burgess S, Nilsen TIL, Damås JK (2023). Association of Genetically Predicted Insomnia With Risk of Sepsis: A Mendelian Randomization Study. JAMA Psychiatry.

